# New morphological and molecular data on the little-known pontellid *Calanopia media* Gurney, 1927 (Crustacea, Copepoda, Calanoida) from the Red Sea, with notes on its diel vertical distribution

**DOI:** 10.3897/zookeys.922.46977

**Published:** 2020-03-25

**Authors:** Mohsen M. El-Sherbiny, Mamdouh A. Al-Harbi

**Affiliations:** 1 Department of Marine Biology, King Abdulaziz University, Jeddah 21589, Saudi Arabia King Abdulaziz University Jeddah Saudi Arabia; 2 Department of Marine Sciences, Suez Canal University, Ismailia 41522, Egypt Suez Canal University Ismailia Egypt

**Keywords:** *Calanopia media*, copepods, pontellid, redescription, Red Sea

## Abstract

As a part of the routine neritic zooplankton collection program in Obhur Creek (central Red Sea, Saudi Arabia), specimens of a pontellid calanoid copepod, *Calanopia media* Gurney, 1927, were observed and studied. Since the original description was rather brief and drawings limited, especially of mouthparts and legs, which were not illustrated and described, the species is here fully redescribed. Red Sea specimens showed considerable variation in the female genital compound somite, the right caudal ramus and leg 5, as well as in the presence of a medial knob ventrally on the male right prosomal corner. DNA sequences of mtCOI of different specimens did not show any significant differences and supported their identity as one species. *Calanopia media* exhibited clear diel vertical migration, with high densities of 106 and 150 ind. m^-3^ during sunset (6:00 pm; UTC+3) and midnight (12:00 am; UTC+3) collections, respectively. However, this species was not observed in other zooplankton collections from the surface to 20 m depth, at 6:00 am and 12:00 pm (UTC+3).

## Introduction

The pontellid (Calanoida) fauna of the Red Sea contains a surprisingly low proportion of the Indo-Pacific fauna, from which it is apparently derived (El-Sherbiny and Ueda 2008; El-Sherbiny 2009). Five genera and 17 pontellid species have been recorded from the Red Sea (Al-Aidaroos et al. 2019; El-Sherbiny and Al-Aidaroos 2017; Razouls et al. 2019), whereas 77 pontellid species are present in the greater Indian Ocean (Razouls et al. 2019). Among the Red Sea pontellids, there are six species of *Calanopia* (Al-Aidaroos et al. 2016; El-Sherbiny and Al-Aidaroos 2017), namely: *C. elliptica* Dana, 1849, *C. minor* A. Scott, 1902, *C. media* Gurney, 1927, *C. kideysi* Ünal & Shmeleva, 2002, *C. thompsoni* A. Scott, 1909 and *C. tulina* El-Sherbiny & Al-Aidaroos, 2017. The original description of *C. media* is rather brief, does not include the cephalic and thoracic limbs, and illustrations are confined to habitus drawings of the female and male in dorsal view, the female urosome, the male right antennule ancestral segments 19 and 20 and the male and female leg 5.

During the examination of plankton, samples collected from the Saudi Arabian waters of the central Red Sea, in Obhur Creek, Jeddah, specimens of what we provisionally called *C. media* were observed. These specimens differ in some respects from Gurney’s (1927) original description. Here, we provide a full description of the species and an account of variability among the Obhur Creek specimens. Comparison with the type specimens held in the Natural History Museum, London (BMNH 1926.2.16.69-88) was carried out, and information on the vertical migration in the water column was provided. In addition, the mitochondrial COI gene of some species from the Red Sea was sequenced and compared with the sequences available in GenBank (NCBI) to determine their affinity.

## Material and methods

Zooplankton samples were collected from Obhur Creek (21°42'32.23"N, 39°5'41.56"E) using a 50 cm diameter plankton net of 150 µm mesh size, towed near the surface for 10 minutes at a speed of about 1–1.5 knots and vertically from 20m depth to the surface on 21 January 2016 at 7:00am, 12:00pm, 6:00pm and 12:00am (UTC+3) local time (sunrise at 7:04am and sunset at 6:05pm; UTC+3). A flowmeter (Hydrobios) was attached to the net mouth for estimating the volume of water filtered. Samples were fixed immediately in 95% alcohol. Subsequently, *Calanopia media* specimens were picked from zooplankton samples collected at midnight. For microscopic examination, specimens were dissected in lactic acid and were observed using bright-field and differential interference microscopy (Nikon DM 6000). Drawings and measurements were made with a camera lucida attached to the microscope and an ocular micrometer. Morphological terminology follows Huys and Boxshall (1991), except for maxillary and maxillipedal appendages, which follow Ferrari and Ivanenko (2008). For scanning electron microscopy, *Calanopia* specimens were washed three times in filtered seawater and clean distilled water, then dehydrated through a 30–100% ethanol series and dried with hexamethyldisilazane. The specimens were mounted on a stub, coated with gold palladium, and observed with a SEM FEI-QUANTA 250.

For genetic analysis, four intact female specimens of *C. media*, three of *C. minor*, one of *C. elliptica* and two of *C. thompsoni* (after accurate morphological identification) were sorted out and the genomic DNA was extracted from individual specimens. A portion of the mitochondrial gene cytochrome oxidase subunit I (mtCOI) was amplified using the universal primers of Folmer et al. (1994). Individual copepods were digested in 400 μl ATL buffer (Qiagen) and 20 μl Proteinase K overnight, in a capped 0.2ml microcentrifuge tube. After digestion, 400 μl of AL buffer was added and DNA extraction continued using Qiagen’s Blood and Tissue kit as per the manufacturer’s instructions. DNA was precipitated in 30 μl AE buffer and mtCOI amplicons were amplified using the PCR primers LCO1490 and HCO2198 (Folmer et al. 1994). The reaction conditions were initial denaturation for 5 min at 95 °C followed by 40 cycles of 94 °C (1 min); 47 °C (2 min); 72 °C (3 min). A final extension at 72 °C for 10 min was undertaken. PCR products were purified using ExoStar (Illustra) and sequencing was carried out in an ABI 3730×l Capillary Sequencer. The machine-read sequences were compiled using Sequencing Analysis (Ver. 3.3, ABI prism) and manually checked for accuracy. Available sequences of *C. thompsoni* were obtained from the NCBI database for comparison. Pairwise distance measures and phylogenetic analyses were conducted using the MEGA X software (Kumar et al. 2018). Ambiguous sites were eliminated from the dataset.

## Results

### Systematics

**Subclass Copepoda Milne-Edwards, 1840**

**Order Calanoida Sars, 1903**

**Family Pontellidae Dana, 1852**

**Genus *Calanopia* Dana, 1852**

#### Calanopia media

Taxon classificationAnimaliaCalanoidaPontellidae

Gurney, 1927

29A12CC7-BCF1-5A3F-B9FA-F2B5C0487D69

**Figure 1.**
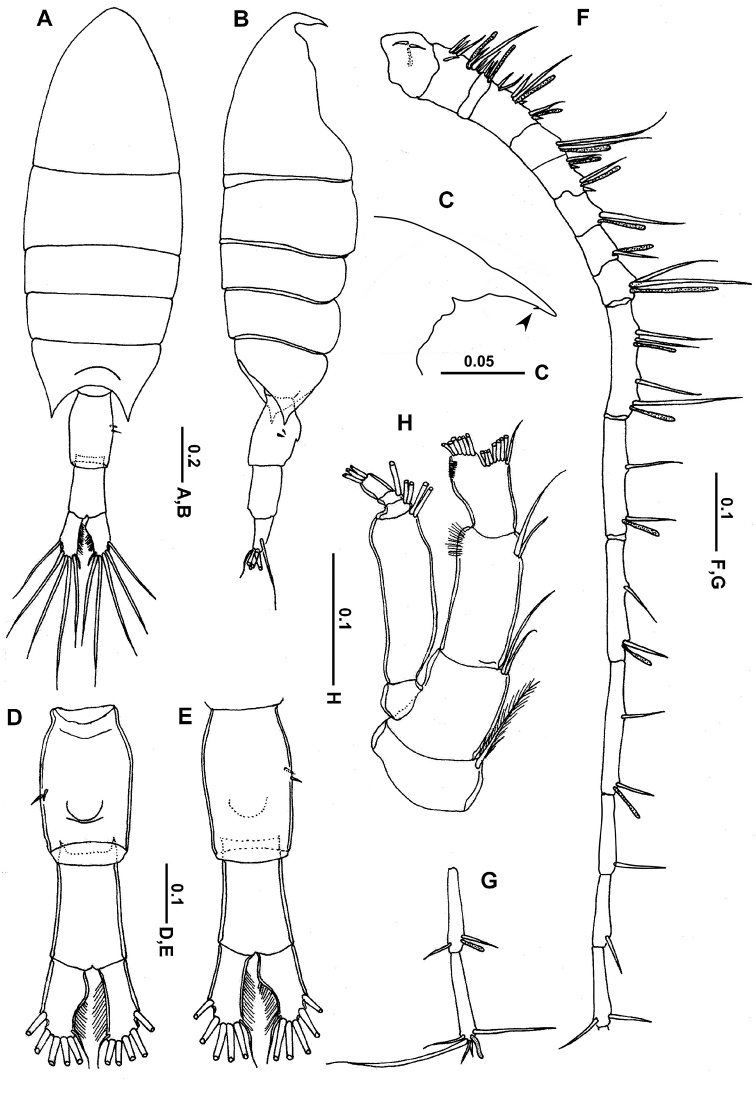
*Calanopia media* female from the Red Sea **A** habitus, dorsal view **B** habitus, lateral view **C** rostrum, lateral view (rudimentary rostral notch indicated by arrow) **D** abdomen, ventral view **E** abdomen, dorsal view **F–G** antennule **H** antenna. Scale bars in mm.

**Figure 2.**
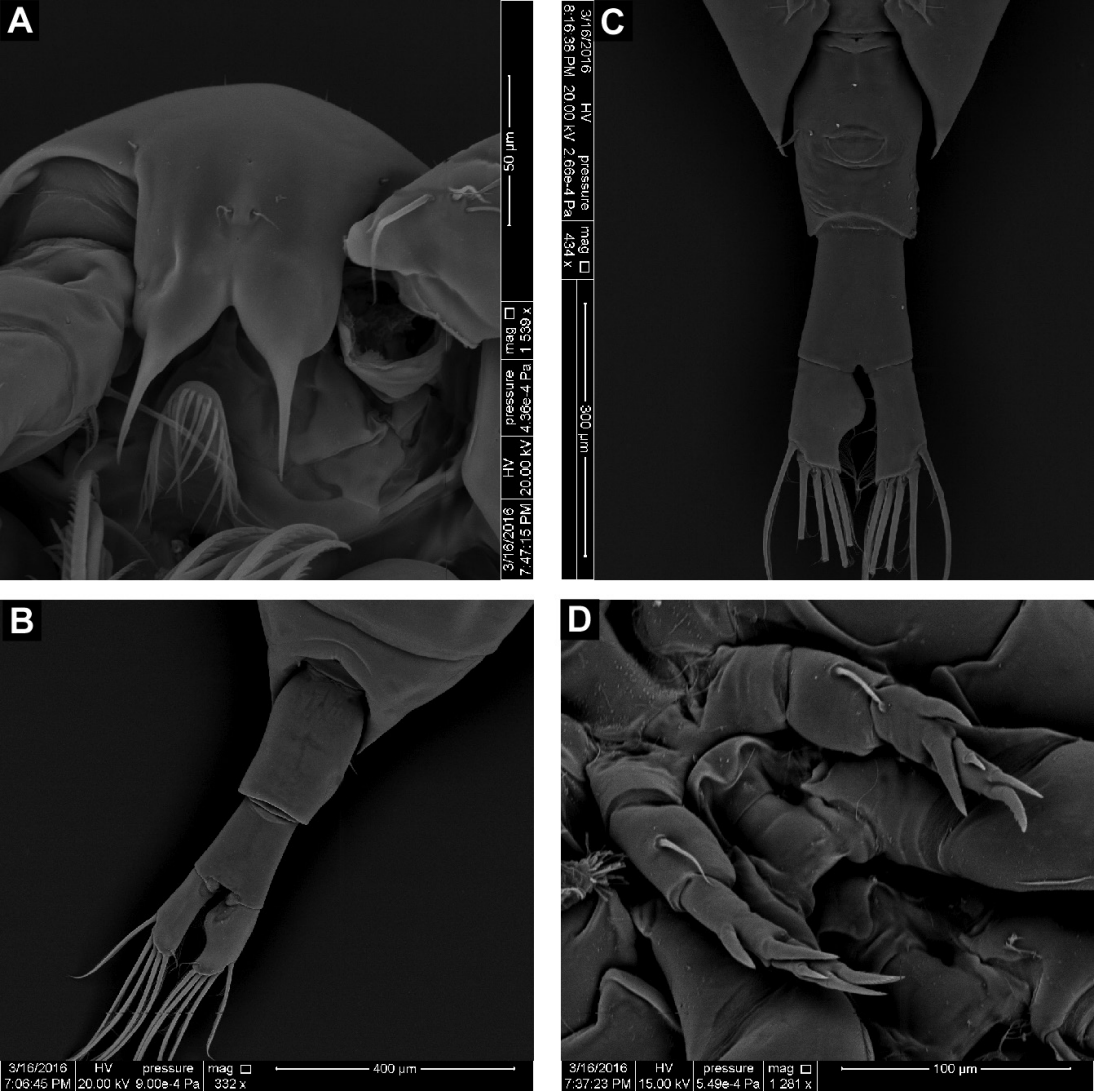
SEM micrographs of *Calanopia media* female from the Red Sea **A** rostrum, ventral view **B** abdomen, dorsal view **C** abdomen, ventral view **D** leg 5, posterior view.

**Figure 3.**
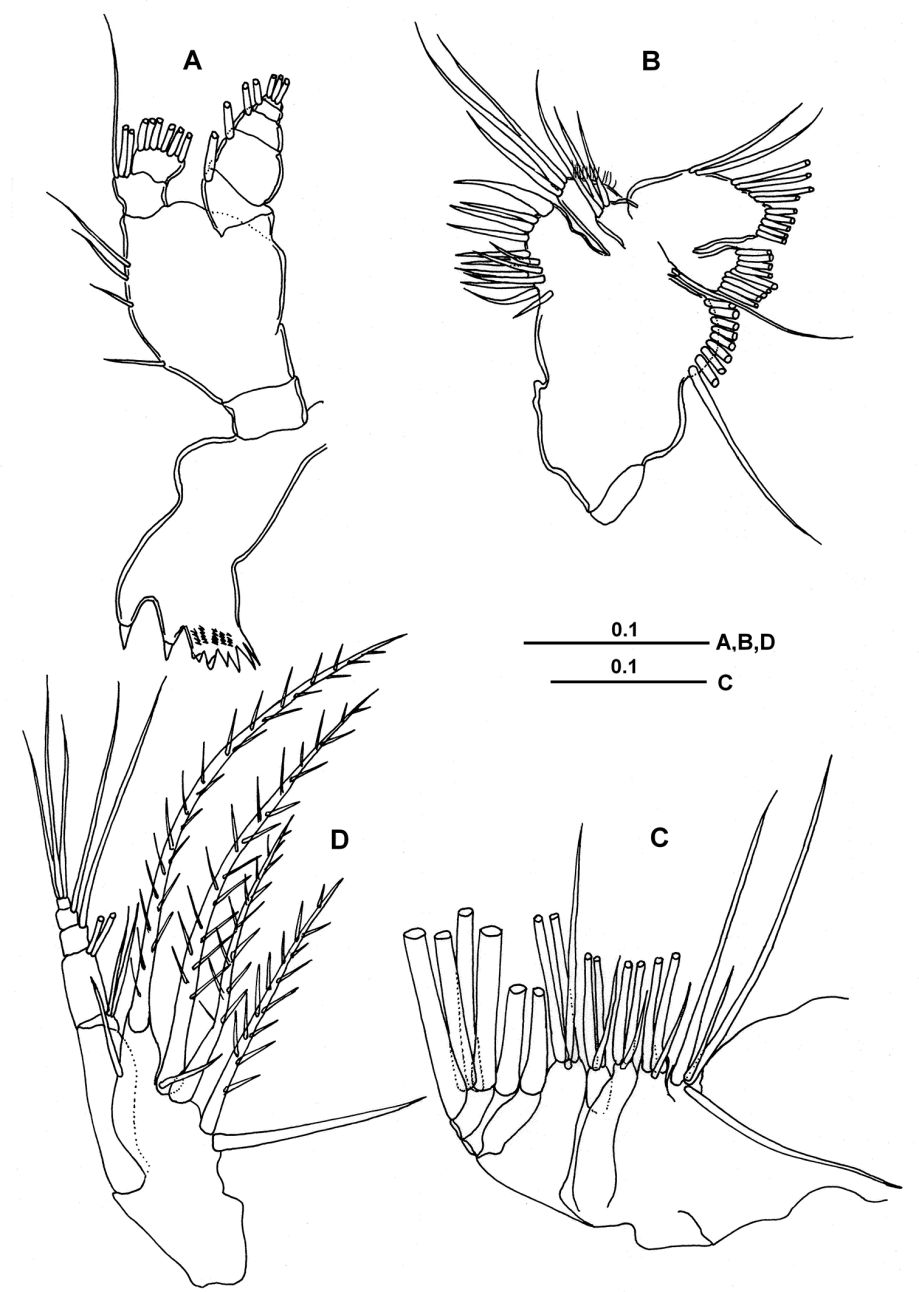
*Calanopia media* female from the Red Sea **A** mandible **B** maxillule **C** maxilla **D** maxilliped. Scale bars in mm.

**Figure 4.**
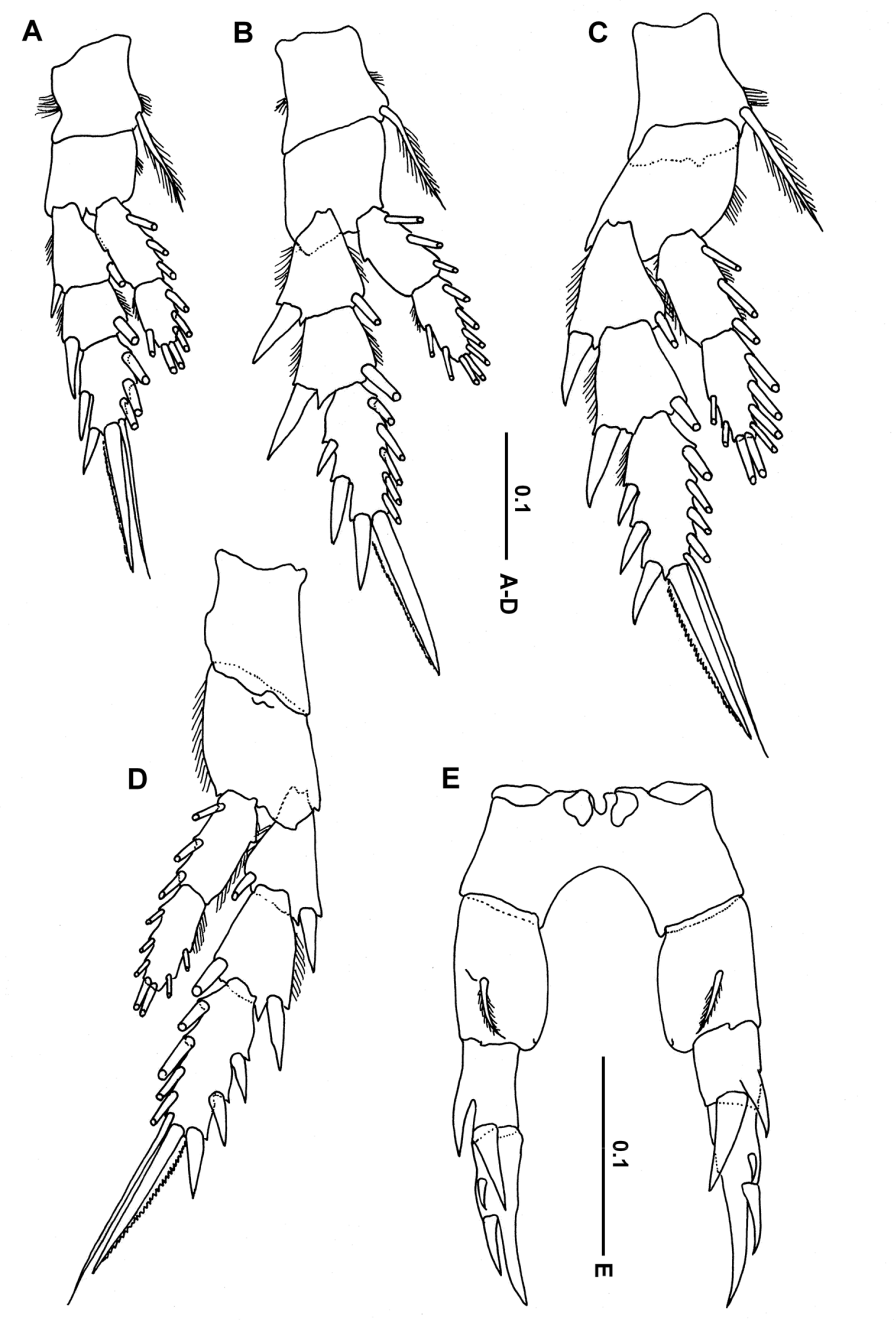
*Calanopia media* female from the Red Sea **A** leg 1, anterior view **B** leg 2, anterior view **C** leg 3, anterior view **D** leg 4, posterior view **E** leg 5, posterior view. Scale bars in mm.

**Figure 5.**
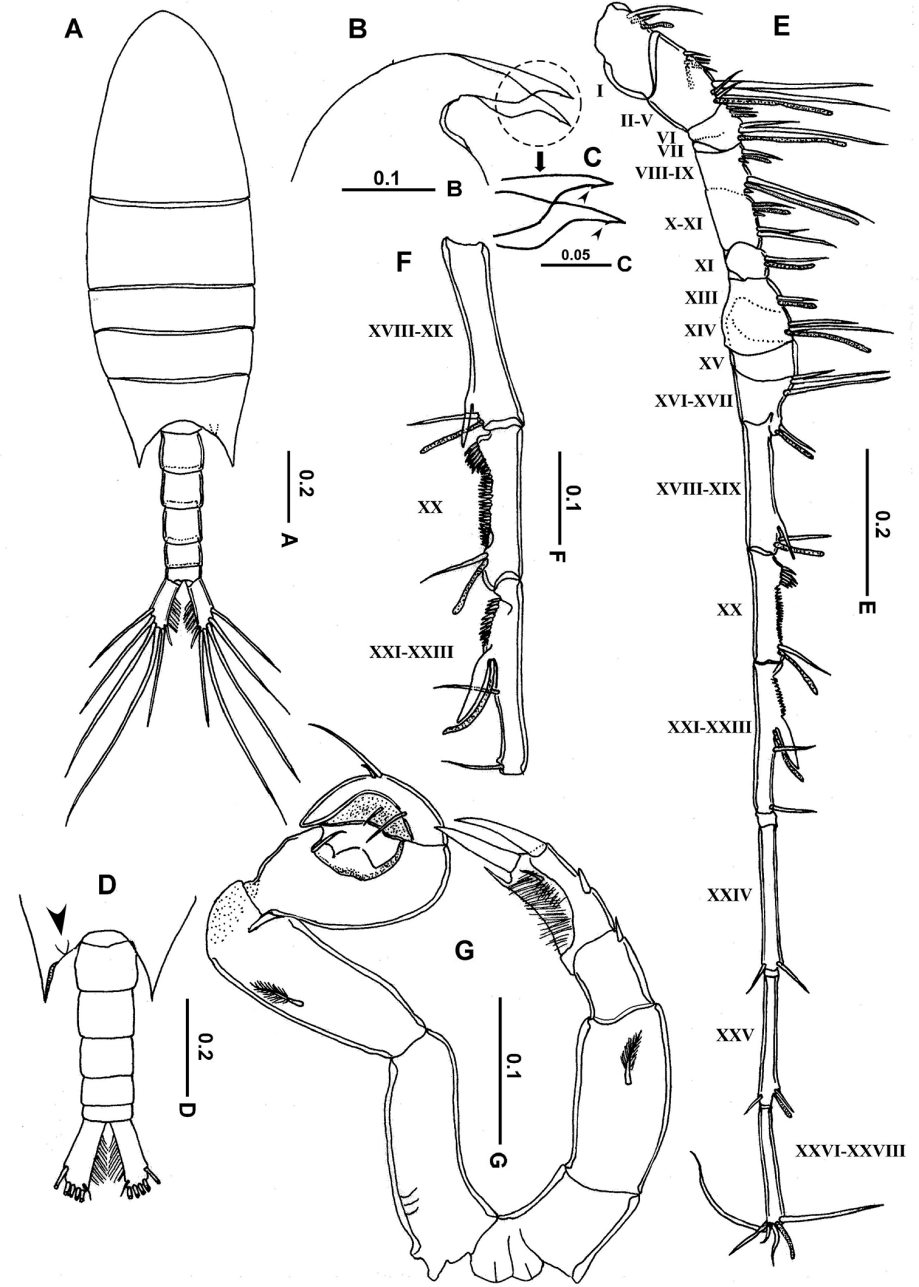
*Calanopia media* male from the Red Sea **A** habitus, dorsal view **B** rostrum, lateral view **C** enlarged rostral filaments (rudimentary rostral notch indicated by arrow) **D** abdomen, ventral view (knob indicated by arrow) **E** right antennule **F** enlarged segments XVIII–XXIII **G** leg 5, posterior view. Scale bars in mm.

**Figure 6.**
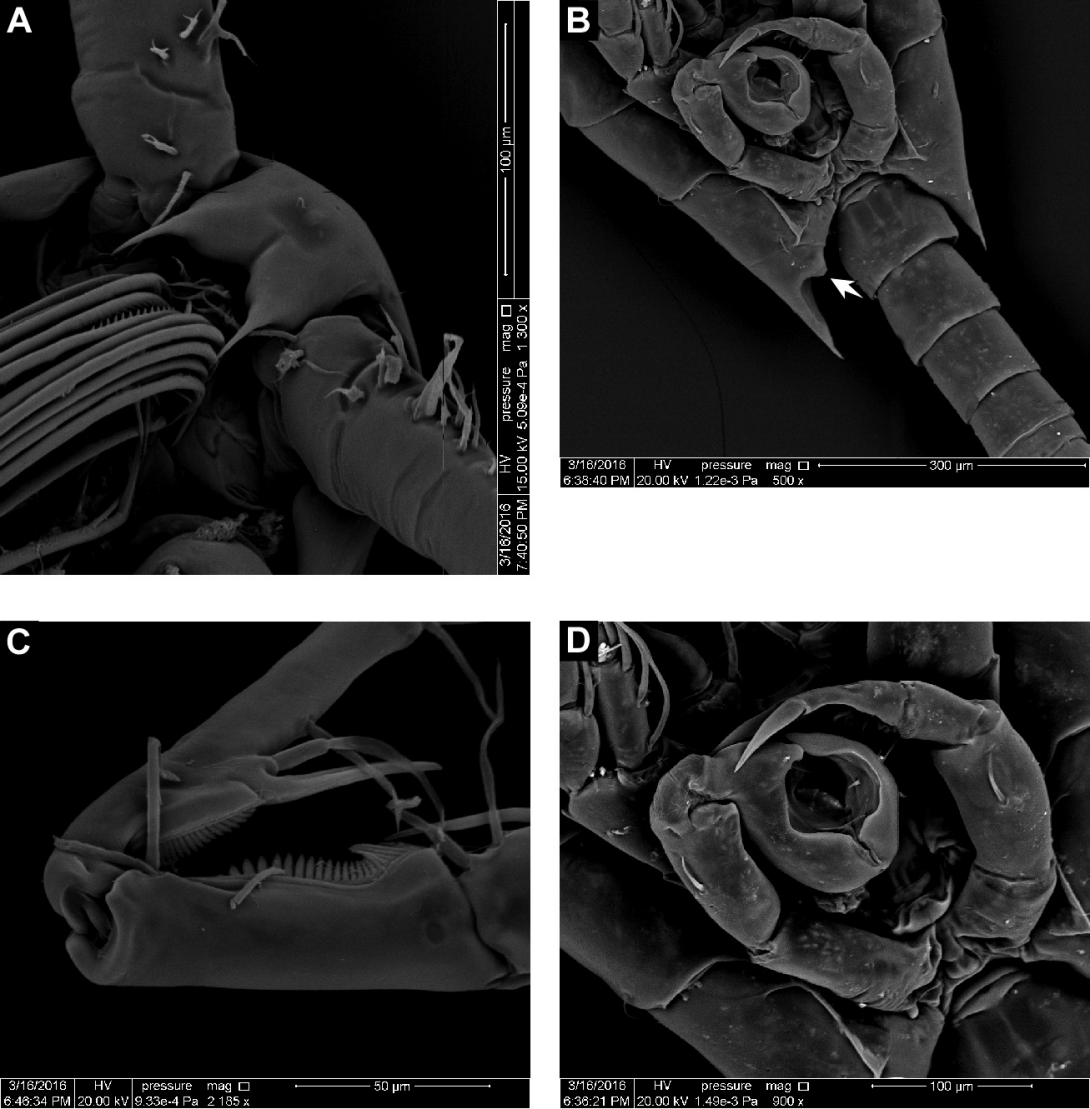
SEM micrograph of *Calanopia media* male from the Red Sea **A** rostrum, ventral view **B** prosomal end with abdomen, ventral view (medial notch indicated by arrow) **C** enlarged segments XX–XXIII **D** male leg 5, posterior view.

Figs 1 , 2 , 3 , 4 , 5 , 6

##### Material examined.

36 females (body length: 1.17–1.32 mm, mean ± SD: 1.25±0.051 mm) and 25 males (body length: 1.10–1.26 mm, mean ± SD: 1.14±0.048 mm); whole specimens in 70% ethanol were deposited in the Natural History Museum, London [Registration number: NHMUK 2018. 1538–1547]. All specimens were collected at Obhur Creek, central Red Sea (21°42'32.23"N, 39°5'41.56"E) on 21 January 2016 by M.M. El-Sherbiny.

##### DNA-barcode.

The mitochondrial gene cytochrome oxidase subunit (mtCOI) sequences were submitted to GenBank (GenBank Accession numbers for *C. elliptica*: MN796254; *C. media*: MN445608–MN445611; *C. minor*: MN796251–MN796253; *C. thompsoni*: MN796255–MN796256).

##### Description.

**Female. *Prosome*** (Fig. 1A, B) elliptical, without lateral hooks; cephalosome and first pedigerous somite completely separated; fourth and fifth pedigerous somites completely fused, with dorsal suture visible; posterior corners of prosome symmetrical, sharply pointed, extending nearly one-third of way along genital compound somite. Rostrum with broad base and pair of rounded lobes, each terminating in a tapering point (Figs 1C, 2A). Urosome (Figs 1D, E, 2B, C) with 2 free somites: genital compound somite symmetrical in dorsal view, with 2 unequal ventral spinules on right side, ventral surface with smooth, evenly rounded operculum located posterior to mid-length (Figs 1E, 2C); second urosomite symmetrical and slightly shorter than genital compound somite; caudal rami asymmetrical; right ramus broader and expanded anteromedially, slightly shorter than left ramus, each ramus carrying 5 plumose setae (II–VI) along distal margin and a reduced seta (seta VII) located on dorsal surface near medial distal angle.

***Antennules*** (Fig. 1F, G) 18-segmented, slightly exceeding end of genital compound urosomite. Fusion pattern and armature elements as follows: ancestral segment I (segment 1) = 2 setae + aesthetasc (ae), II–VI (2) = 8 + ae, VII (3) = 2 + ae, VIII–X (4) = 7 (2 spiniform) + 2ae, XI (5) = 2 + ae, XII–XIII (6) = 4 (2 spiniform) + 2ae, XIV (7) = 1 +ae, XV (8) = 1 + ae, XVI (9) = 2 + ae, XVII–XVIII (10) = 4 + 2ae, XIX (11) = 2 + ae, XX (12) = 2 + ae, XXI (13) = 2 + ae, XXII (14) = 1, XXIII (15) = 1, XXIV (16) = 1 + 1, XXV (17) = 1 + ae+ 1, XXVI–XXVIII (18) = 5 + ae.

***Antenna*** (Fig. 1H) biramous; coxa with plumose seta distomedially; basis carrying 2 subequal plumose setae at distomedial angle; exopod 5-segmented, second segment longest with setal formula of 0, 2, 2, 1, 3. Endopod 2-segmented, first endopodal segment with 2 subequal lateral setae distally and furnished with fine setules distolaterally; second endopodal segment armed with 8 and 6 setae on proximal and distal lobes, respectively, laterodistal border with row of posterior spinules.

***Mandible*** (Fig. 3A). Gnathobase with eight teeth on cutting edge, third and fourth ventralmost teeth bicuspidate; patches of dagger-like spinules arranged at base of third to sixth ventralmost teeth; mandibular palp basis with 4 setae; endopod 2-segmented, first and second segments carrying 3 and 6 setae, respectively; exopod 5-segmented, first to fourth segments each with one seta and fifth segment with 3 setae.

***Maxillule*** (Mx1) (Fig. 3B). Praecoxal endite well developed and extended distally with 9 marginal and 4 posterior setae; coxal exite bearing 9 setae along distal margin; coxal endite with 3 setae; basal exite with long seta, proximal and distal endites with 3 and 2 setae, respectively. Exopod 1-segmented, with a total of 9 terminal setae. Endopod fused to basis, bearing 4 medial and 5 terminal setae.

***Maxilla*** (Mx2) (Fig. 3C). Praecoxal endite of syncoxa with 4 setae; proximal and distal coxal endites bearing 3 setae each; proximal and distal basal endites with 3 and 3 setae, respectively; endopod 3-segmented, with setal formula of 1, 1, 4.

***Maxilliped*** (Mxp) (Fig. 3D). Praecoxa and coxa completely fused, syncoxa with three endites carrying 2, 3, 2 setae on proximal, middle and distal endites, respectively; basal endite with 2 distal setae; endopod 4-segmented, first endopodal segment long, with 2 setae distally; other three endopodal segments shorter, bearing 1, 1 and 3 setae, respectively.

***Legs*** 1–4 as in other members of the genus, with 3-segmented exopods and 2-segmented endopods as well as lateral spines with serrated hyaline margins (Fig. 4A–D): coxa of legs 1 to 3 bearing one medial seta and a patch of fine setules; coxa of leg 4 without medial seta. Seta and spine formula as follows (spines, Roman numerals; setae, Arabic numerals):

**Table:** 

	Coxa	Basis	Exopod			Endopod	
			1	2	3	1	2
Leg 1	0–1	0–0	I–1	I–1	II, I, 4	0–3	1, 2, 3
Leg 2	0–1	0–0	I–1	I–1	III, I, 5	0–3	2, 2, 4
Leg 3	0–1	0–0	I–1	I–1	III, I, 5	0–3	2, 2, 4
Leg 4	0–0	0–0	I–1	I–1	III, I, 5	0–3	2, 2, 3

Leg 5 (Fig. 4E) asymmetrical but with same number of spines and processes; coxa and intercoxal sclerite completely fused; right basis broader and slightly shorter than left basis, each with one posterior plumose seta; exopod 2-segmented; first exopodal segment of right leg shorter than that of left leg; with 1 lateral fused process and 1 strong spine distally; second exopodal segment of right leg slightly longer than that of left leg, extending into tapering process fused to its segment, with 2 lateral articulated spines (proximal one smaller).

**Male. *Prosome*** (Fig. 5A) 2.1 times as long as urosome; cephalosome and first pedigerous somite completely separated; fourth and fifth pedigerous somites completely fused (Figs 5A, 6B); rostrum as in female (Fig. 5 B, C); posterior corners of prosome slightly asymmetrical (right one slightly longer than left), with a sharp triangular process directed posteriorly and with a distinct ventral knob or process on its right medial margin, which cannot be seen in dorsal view (Figs 5D, 6B). Urosome composed of 5 free somites, genital somite with genital aperture located ventrolaterally on posterior left side margin; second urosomite longer than other somites; anal somite shorter than preceding somite; caudal rami symmetrical, 2.2 times longer than wide, each ramus with 6 setae (II–VI) and seta VII small, inserted in ventrodistal medial margin.

***Antennule*** (Figs 5E, F, 6C) geniculate on right side, left one similar to that of female (except for second segment, which carries longer posterior setae): right one indistinctly 17–segmented, segments 3–4 incompletely fused ventrally, segments 5–6 and 7–8 completely fused dorsally, segment 13 with long denticles on proximal 1/4 and short denticles that extend to distal fourth part, segment 14 tooth ridge possessing triangle denticles proximally, which extend back to distally-directed spure-like process, armature as follows: ancestral segment I (segment 1) = 2 setae + aesthetasc (ae), II–V (2) = 8 + 2ae, VI (3) = 2 + ae VII (4) = 2 + ae, VIII–IX (5) = 4 (2 spiniform) + 2ae, X–XI (6) = 4 (1 spiniform) + ae, XII (7) = 1 + ae, XIII (8) = 1 + ae, XIV (9) = 2+ ae, XV (10) = naked, XVI–XVII (11) = 3 + 2 ae, XVIII–XIX (12) = 1+ process] + ae, XX (13) = 1+ ae, XXI–XXIII (14) = 2 + process +ae, XXIV (15) = 1 + 1, XXV (16) = 1+ ae + 1, XXVI–XXVIII (17) = 5 + ae.

Antenna, mouthparts and legs 1–4 as in female. Leg 5 (Figs 5G, 6D) uniramous, asymmetrical; coxae and intercoxal sclerite completely fused. Left leg basis carrying 1 plumose seta posteriorly near two-thirds of its length; exopod 2-segmented, first segment shorter than basis (0.46 times) with small laterodistal spine; second segment nearly 1.35 times as long as first one, bearing 3 articulated spines (2 stout apically and one small laterally), medial hirsute margin with one distal fused spine. Right leg 5 (Figs 5G, 6D), longer than left; basis slightly longer than coxa, carrying one posterior plumose seta; right exopod 2-segmented, first segment with small thumb-like process located at approximately one-third of segment length, with small seta near base of thumb, lateral margin concave with bilobed flap-like process; second exopodal segment approximately 0.7 as long as first exopod segment, curved at about mid-length and bluntly rounded distally, bearing 2 setae in depression (one proximal and one central) and 2 unequal outer setae at mid-length (proximal one longer than distal).

##### Variations.

On the ventral surface of the female genital compound somite of some specimens, a small fold in the cuticle may be found on the right or left side. Also, the degree of anteromedial expansion of the female right caudal ramus varies among specimens. The anteromedial expansion of the female right caudal ramus was present in most of the specimens collected from the study area (about 90% of the population), and sometimes the degree of this expansion varied greatly among specimens. In some specimens, the right caudal ramus had a concave or straight medial margin. Moreover, the ventral knob on the right side of the male prosome posterior corners varies in size.

##### Remarks.

We compared our specimens with the paratypes deposited at the Natural History Museum, London (BMNH 1926.2.16.69-88), and concluded that our specimens are *C. media*. Both our specimens and the paratypes shared most of the diagnostic features of the species, such as: the shape of the fifth pediger, the presence of 2 ventral spinules on the right side of the female genital compound somite, and the structure of both female and male leg 5. However, the asymmetry of female leg 5 (right leg basis broader and slightly shorter than left, first exopodal segment of right leg shorter than that of left leg and second exopodal segment of right leg slightly longer than on left leg) and the presence of a ventral knob on the right side of the prosome were probably overlooked in the original description by Gurney (1927). Nevertheless, our specimens differ in the asymmetry of the caudal rami, of which the right ramus is broader and expanded anteromedially, and slightly shorter than the left one.

##### Distribution.

*Calanopia media* was originally described from the Suez Gulf and the southern part of the Suez Canal (Gurney 1927) during the CAMBRIDGE Expedition. Subsequently, Pesta (1941) collected this species during the POLA Expedition in the southern Red Sea (15°26'12"N, 40°05'24"E). In 1956, Rose recorded this species from the Vietnamese waters. Later, it was recorded from the Levantine Basin by Berdugo (1968) and Lakkis (1984) and considered to be a Lessepsian migrant species. In the present study, *C. media* was found in considerable abundance (106 ind. m^-3^) in samples collected at sunset (6:00 pm; UTC+3), with the highest densities at midnight (150 ind. m^-3^). Copepodid stages were relatively low, constituting 9 and 5% of the population at sunset and midnight, respectively. It was completely absent from near the surface in morning and midday samples. The sex ratio (males/females) of *C. media* varied between 0.46 and 0.54 at 6:00 pm and 12:00 am (UTC+3), respectively.

##### Molecular diversity.

A 624-bp region of the mtCOI was obtained for four female individuals of *C. media*, which varied in the degree of anteromedial expansion of the female right caudal ramus in specimens collected from Obhur Creek, central Red Sea. Results showed that the four analyzed specimens have nearly identical mtCOI sequences, with a distance ranging between 0.013 and 0.016 based on the pairwise distance method and Kimura 2 parameter model. The intraspecific variation in the mtCOI sequences of the other Red Sea species, *C. minor* and *C. thompsoni*, were 0.000 and 0.002, respectively.

**Figure 7.**
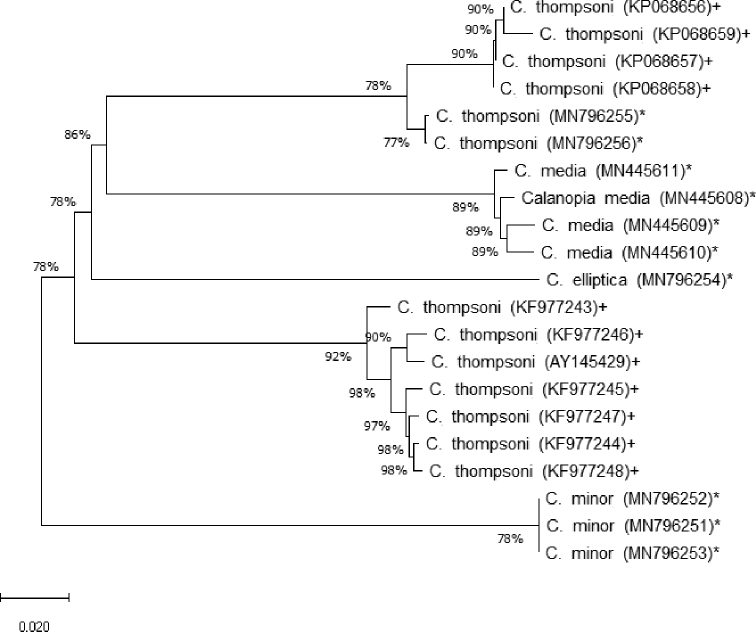
Neighbor Joining phylogenetic tree based on the mtCOI genes of *Calanopia elliptica*, *C. media*, *C. minor* and *C. thompsoni* from the Red Sea (indicated by *). *Calanopia thompsoni* sequences (indicated by +) from GenBank were used for comparative analysis.

Moreover, in the current analysis, sequences were obtained for three other *Calanopia* species collected from the study area (*C. elliptica*, *C. minor* and *C. thompsoni*) and sequences of one species (*C. thompsoni*) were obtained from NCBI. The mtCOI sequences of *Calanopia* species (i.e., *C. elliptica*, *C. media*, *C. minor* and *C. thompsoni*) from the Red Sea differ between 21.3% and 29.4% (Table 1 and Fig. 7). A Neighbor Joining phylogenetic analysis using the Kimura 2 parameter model showed that *C. media* was clearly distinct from its congeneric species (Fig. 7). Concerning *C. thompsoni*, the only sequenced mtCOI in NCBI, it is clear that the average distance between Red Sea specimens and Indian Ocean specimens (KP068656–KP068659) was 0.035 (0.030–0.042), whereas for the China seas’ specimens it was 0.201 (0.193–0.211).

**Table 1.** Pairwise distances for mtCOI sequences between *Calanopia elliptica*, *C. media*, *C. minor* and *C. thompsoni* from the Red Sea (indicated by *). *Calanopia thompsoni* sequences from GenBank (indicated by +) were used for comparative analysis.

No.	Species	1	2	3	4	5	6	7	8	9	10	11	12	13	14	15	16	17	18	19	20
1	*C. media* (MN445608)*																				
2	*C. media* (MN445609)*	0.016																			
3	*C. media* (MN445610)*	0.013	0.016																		
4	*C. media* (MN445611)*	0.013	0.013	0.013																	
5	*C. minor* (MN796251)*	0.289	0.279	0.291	0.282																
6	*C. minor* (MN796252)*	0.288	0.279	0.290	0.282	0.000															
7	*C. minor* (MN796253)*	0.288	0.279	0.290	0.282	0.000	0.000														
8	*C. elliptica* (MN796254)*	0.263	0.278	0.260	0.263	0.294	0.293	0.293													
9	*C. thompsoni* (MN796255)*	0.227	0.238	0.238	0.233	0.270	0.269	0.269	0.232												
10	*C. thompsoni* (MN796256)*	0.231	0.242	0.241	0.236	0.267	0.267	0.267	0.235	0.002											
11	*C. thompsoni* (KP068656)+	0.213	0.225	0.223	0.218	0.276	0.275	0.275	0.235	0.034	0.036										
12	*C. thompsoni* (KP068657)+	0.211	0.223	0.220	0.216	0.273	0.272	0.272	0.232	0.032	0.034	0.002									
13	*C. thompsoni* (KP068658)+	0.211	0.223	0.220	0.216	0.275	0.275	0.275	0.235	0.030	0.033	0.003	0.002								
14	*C. thompsoni* (KP068659)+	0.223	0.235	0.233	0.228	0.290	0.290	0.290	0.248	0.040	0.042	0.008	0.010	0.008							
15	*C. thompsoni* (KF977243)+	0.221	0.224	0.226	0.215	0.253	0.252	0.252	0.225	0.195	0.193	0.229	0.227	0.226	0.238						
16	*C. thompsoni* (KF977244)+	0.240	0.242	0.244	0.233	0.255	0.255	0.255	0.244	0.208	0.206	0.241	0.239	0.239	0.251	0.022					
17	*C. thompsoni* (KF977245)+	0.235	0.238	0.240	0.228	0.258	0.258	0.258	0.244	0.198	0.196	0.234	0.232	0.231	0.244	0.026	0.009				
18	*C. thompsoni* (KF977246)+	0.231	0.233	0.235	0.224	0.264	0.263	0.263	0.239	0.200	0.198	0.224	0.222	0.222	0.234	0.022	0.021	0.018			
19	*C. thompsoni* (KF977247)+	0.242	0.245	0.247	0.235	0.252	0.252	0.252	0.239	0.211	0.208	0.239	0.236	0.236	0.248	0.022	0.006	0.009	0.021		
20	*C. thompsoni* (KF977248)+	0.233	0.235	0.237	0.226	0.255	0.255	0.255	0.244	0.203	0.201	0.243	0.240	0.240	0.252	0.021	0.004	0.010	0.019	0.007	
21	*C. thompsoni* (AY145429)+	0.231	0.233	0.235	0.224	0.267	0.266	0.266	0.233	0.205	0.203	0.221	0.219	0.219	0.231	0.026	0.018	0.018	0.011	0.018	0.020

## Discussion

In his original description of *C. media*, Gurney (1927) provided drawings only for the whole body and leg 5 of both sexes, as well as of the female urosome and the geniculate part of the male right antennule (segments XX and XXI), and compared it with *C. elliptica*. His description was brief and focused mainly on the female genital compound somite and female leg 5. For the male, he only mentioned that the major differences between *C. media* and *C. elliptica* lay in a lack of serrations on the second exopodal segment of right leg 5. In his words, “Fifth legs closely resembling those of *C. elliptica*, but without the scalloped edge to the broad subterminal joint of the right leg”. Re-examination of *C. media* from the study area allowed us to more accurately describe this species. The original characters reported by Gurney (1927) are given in brackets: 1) the female genital compound carries one or two spinules on the right side (2 spinules); 2) the female caudal rami are asymmetrical, the right one is expanding anteromedially (symmetrical); 3) the female leg 5 is slightly asymmetrical, with the left leg slightly shorter than the right due to shortness of both the basis and first exopodal segment (mentioned as symmetrical by Gurney, but from his drawings the first exopodal segment is longer on the right side and also the right second exopod segment is longer than the left one); 4) the male prosomal corners project posterolaterally into asymmetrical pointed processes, the right one of which is slightly longer than the left, with a distinct ventral knob on its medial margin, invisible in dorsal view (symmetrical); and 5) the second exopod segment of the male right leg 5 carries one seta medially, which is lacking in Gurney’s description. Moreover, Pesta (1941), in his description from the southern Red Sea, mentioned the lack of spinules on the genital compound somite, while the female leg 5 exopod proportions differed slightly from Gurney’s description (in Pesta’s description, the second exopod segment is 1.1 times as long as the first exopod segment) and the second distal spine on the second exopod segment is shorter than in Gurney’s description.

In addition to the variability noted in the caudal rami of *C. media*, variation has been reported in *C. sewelli* Jones & Park, 1967, collected from Marquesas, central Pacific, in which the right ramus was sometimes longer and with a concave medial margin. Moreover, asymmetry of the female caudal rami has been noted in *C. asymmetrica* Mulyadi & Ueda, 1996 collected from Indonesian waters, where the right ramus was much longer than the left one and expanded posteriorly. This asymmetry was found also in *C. australica* Bayly & Greenwood, 1966 collected from Moreton Bay, Australia, in which the left ramus was longer than the right. Asymmetry of the male prosomal points in *C. media* is similar to that in *C. sarsi* C.B. Wilson, 1950 collected from Fiji waters and *C. tulina* (El-Sherbiny and Al-Aidaroos 2017) described from the Red Sea.

*Calanopia media* is closely related to *C. tulina* from the central Red Sea, but they can be distinguished from each other by the characters listed in Table 2. The most distinctive characters of *C. media* are: 1) the presence of 2 ventral spinules on the right side of the female genital compound somite, 2) the extreme asymmetry of the caudal rami of the female, 3) the presence of 2 long articulated and 1 short fused terminal spines on the second exopodal segment of the male left leg 5, 4) the first exopodal segment of male right leg 5 with a small thumb located approximately at one-third of the segment length and with the semicircular processes on a flap on its lateral part, and 5) the second exopodal segment of the male right leg 5 shorter than the first exopodal segment, which is curved at mid-length with a blunt apex and 2 medial setae within a shallow medial depression.

**Table 2.** Comparative list of characters of *Calanopia media* and *C. tulina*. The characters of *C. tulina* are taken from the original description by El-Sherbiny and Al-Aidaroos (2017).

	*Calanopia media*	*Calanopia tulina*
**Female**		
Rostral points	With rudimentary subterminal notch	With small subterminal notch
Genital compound somite	With two spinules on right side	Without any spinules
Caudal rami	Asymmetrical, right one slightly shorter than left and expanded antero-medially (varies between individuals)	Asymmetrical, right one slightly shorter than left
Leg 5	Asymmetrical; right basis boarder and shorter than right; right first and second exopodal segments shorter than on left	Asymmetrical; left leg slightly shorter than right; left basis, first and second exopodal segment shorter than on right
**Male**		
Rostral points	With rudimentary subterminal notch	With small subterminal notch
Posterior prosome	Asymmetrical, right point slightly longer, with small ventral knob on medial margin	Asymmetrical, right point wider and slightly longer, with distinct knob on medial margin
Second exopodal segment of left leg 5	With 3 articulated spines (2 stout and terminal, and one small and lateral)	With 2 relatively long curved, terminal medially-serrated articulated spines and 1 lateral spine directed medially
First exopodal segment of right leg 5	With small thumb-like process located at one-third of segment length, with bilobed flap-like process	With very small rounded-tip thumb-like process located nearly mid-length, central medial part smooth, not bilobed
Second exopodal segment of right leg 5	Curved at mid-length with 2 setae on concave surface, and with 2 unequal setae at mid-length on convex surface	Curved at two-thirds length, with 2 setae on concave surface, and 2 unequal setae at mid-length on convex surface.

In our study, *C. media* exhibited a clear diel vertical migration (DVM). Sunset ascend and sunrise descent were performed at very low light intensities. This pattern is known as the nocturnal or normal DVM (Forward 1988). Similar observations were made in previous studies in the southern part of the Suez Canal and the southern Red Sea by Gurney (1927) and Pesta (1941), respectively, who reported that *C. media* was found in high abundance in the coastal waters mainly in the night samples. This pattern was recorded also for *C. americana* F. Dahl, 1894 in the eastern Gulf of Mexico (Turner et al. 1979) and in the Brazilian waters (Pessoa et al. 2014). This DVM may be performed to avoid UV radiation, light and predators, as well as for availability of food and favorable temperature (Bollens and Frost 1989; Lampert 1989; Andersen and Nival 1991; Pearre 2003; Cohen and Forward 2005). Generally, the light intensity in Obhur Creek drops steeply below 5 m depth, and very little light penetrates into waters below 15 m (Al-Aidaroos et al., unpublished data). Following the same trend, UVB radiation decreased by 74.15% at 5 m depth, while only 2% reached 15 m depth (Duarte et al., unpublished data). This suggests that *C. media* is avoiding high temperature, high light intensity and/or visual predation during the day and coming up to the surface layers for feeding during night-time. This species was observed to be abundant in night collections by light trap from a shallow coral reef in the central Red Sea (unpublished data). This might be attributed to its benthopelagic behavior, which is clearly visible in terms of the stout, large outer spines on its legs (1–4), as reported in the benthopelagic copepod genus *Platycopia* (Ohtsuka et al. 1998).

Morphology can be considered as a fundamental method for copepod species identification. However, some pontellid species display considerable intraspecific morphological variations in the female genital compound somite, caudal rami and fifth leg (Jeong et al. 2014). Such differences might be sufficient to prove the presence of a new species, as it is the case for some centropagoid species (e.g. Sakaguchi and Ueda 2010; Soh et al. 2012). The mtCOI gene was proposed as a unique tool for copepod identification with ‘barcoding’ (Bradford et al. 2010; Blanco-Bercial et al. 2014), which can verify the species identity within morphologically varying pontellid specimens. Within Crustacea, the level of genetic variation between congeneric species is 17.16%, while the level of intraspecific variability is 0.46% (Costa et al. 2007). Moreover, variation between calanoid copepod species varied between 13–22%, 17.6–26.7% and 21–23% in previous studies by Bucklin et al. (1999), Eyun et al. (2007) and Soh et al. (2013), respectively. In this study, the intraspecific variation in the COI sequences from the Red Sea specimens of *C. media*, *C. minor* and *C. thompsoni* would confirm the hypothesis of Costa et al. (2007). The *Calanopia* species nucleotide sequences collected from GenBank indicate no genetic differentiation between the specimens of *C. thompsoni* from the Red Sea and the Indian Ocean. In contrast, the nucleotide data of *C. thompsoni* from the Red Sea revealed considerable variations with specimens collected from the China seas, indicating that the two populations are genetically different. This high level of deviation between both populations can by supported by the allopatric speciation hypothesis of Carpenter and Springer (2005), which states that in the Pleistocene the migration of marine organisms from the West Pacific Ocean to the Indian Ocean seems to be blocked by an ecological vicariant. Moreover, sequences included herein from the Red Sea are the first barcodes for these species, and it will be useful in future pontellid barcode studies.

During the last two decades, six species of pontellids have been originally described as new species or were first recorded from the Red Sea: *Calanopia kideysi* by Ünal and Shmeleva (2002), *Labidocera boxshalli* El-Sherbiny & Ueda, 2010 by El-Sherbiny and Ueda (2010), *Calanopia tulina* by El-Sherbiny and Al-Aidaroos (2017), *Pontella princeps* Dana, 1849 by El-Sherbiny (2009), *Labidocera karachiensis* Fazal-Ur-Rehman, 1973 by El-Sherbiny and Ueda (2008), and *Calanopia thompsoni* by Al-Aidaroos et al. (2016). However, the diversity of this family in the Red Sea is rather low (17 species) compared with that of the Indian Ocean (77 species as reported by Razouls et al. 2019), from which the Red Sea fauna has originated (Al-Aidaroos et al. 2019). This low number can be attributed to the characteristic euneustonic or facultative neustonic nature of this group (Mauchline 1998). Thus, inappropriate sampling methods and sampling time and/or limited sampling effort might have resulted in an underestimation of the fauna of Pontellidae. Therefore, this study emphasizes the necessity of understanding the diversity and distribution of pontellid copepods in the Red Sea and their mode of life.

### Key to species of *Calanopia* recorded in the Red Sea

**Females**

**Table:** 

1	Leg 5 exopod one-segmented	**2**
–	Leg 5 exopod two-segmented	**3**
2	Genital compound somite shorter than second urosomite; exopodal segment of leg 5 with two small lateral spines and one long medial spine (longer than the segment itself)	***C. minor***
–	Genital compound somite nearly equal to second urosomite; exopodal segment of leg 5 with two small lateral spines and one medial spine (shorter than the segment itself)	***C. kideysi***
3	Cephalic lateral hooks absent	**4**
–	Cephalic lateral hooks present	***C. thompsoni***
4	Genital compound somite naked, without lateral spinules	**5**
–	Genital compound somite with two lateral spinules on the right side	***C. media***
5	Leg 5 asymmetrical; left leg distinctly longer than right one	***C. elliptica***
–	Left leg 5 slightly asymmetrical; right leg slightly shorter than left one	***C. tulina***

**Males (*C. kideysi* not included since the adult male is unknown)**

**Table:** 

1	Cephalic lateral hooks absent	**2**
–	Cephalic lateral hooks present	***C. thompsoni***
2	Left leg 5 shorter than right one; basis of left leg 5 not swollen proximally	**3**
–	Left leg 5 longer than right one; basis of left leg 5 swollen proximally and produced into a small curved tooth	***C. minor***
3	Second urosomite with one acuminate-tip spinose process postero-laterally on right side; first exopodal segment of right leg 5 with three strong blunt teeth and second exopodal segment claw-like with three small pointed teeth	***C. elliptica***
–	Second urosomite naked; first and second exopodal segments of right leg 5 without any teeth	**4**
4	Second exopodal segment of left leg 5 with three articulated spines (two stout terminally, one small laterally); second exopodal segment of right leg 5 curved at mid-length with a relatively short spine laterally	***C. media***
–	Second exopodal segment of left leg 5 with two relatively long curved, terminal, medially-serrated spines and one lateral spine directed medially; second exopodal segment of right leg 5 curved at two-thirds of length, with relatively long spine laterally	***C. tulina***

## Supplementary Material

XML Treatment for Calanopia media
